# Unraveling the Disease Mechanisms of Neuropathic Pain Through Constructing miRNA–mRNA Networks Based on a Rat Model

**DOI:** 10.1002/brb3.70812

**Published:** 2025-10-08

**Authors:** Lingqi Yao, Ming Liu

**Affiliations:** ^1^ Department of Anesthesiology The First People's Hospital of Xiaoshan District Hangzhou Zhejiang China

**Keywords:** bioinformatics, biomarkers, differential expression, microRNA‐messenger RNA (mRNA), neurogenic pain

## Abstract

**Background:**

Neuropathic pain (NP) lacks clear biomarkers and effective treatment methods. We aimed to identify important genes and microRNA (miRNA)–messenger RNA (mRNA) regulatory network in NP to elucidate the underlying mechanism of NP using bioinformatics analysis combined with an animal model.

**Methods:**

Two NP‐related gene expression datasets were obtained from the Gene Expression Omnibus (GEO) database. Differentially expressed genes (DEGs) between NP and controls were identified using the limma package. Protein–protein interaction (PPI) and miRNA–mRNA‐disease networks were constructed to investigate the interactions among genes and miRNAs. Kyoto Encyclopedia of Genes and Genomes (KEGG) was performed to investigate the biological functions of DEGs in NP. Additionally, to further confirm the expression and the functions of hub genes, a chronic constriction injury (CCI) NP rat model was established, and *C1qb* knockdown treatment was performed by transfection of sh‐C1qb.

**Results:**

A total of 108 common DEGs (94 upregulated and 14 downregulated) were identified related to the pathogenesis of NP. Five hub genes (*Ptprc*, *C1qb*, *Aif1*, *Fcgr2b*, and *Ccl2*) were selected in the PPI network. KEGG analyses unveiled that the five hub genes were primarily involved in immune regulation and neuroinflammation especially NF‐κB signaling pathway. miRNA–mRNA‐disease network analysis revealed 160 miRNAs associated with the five hub genes, and 25 miRNAs (including miR‐124‐3p, miR‐128‐3p, and miR‐369‐3p) were regulated to *Ptprc*, *C1qb*, *Fcgr2b*, and *Ccl2* in NP. Moreover, the expressions of Aif1, Ptprc, C1qb, Fcgr2b, and Ccl2 were increased in blood, spinal cord, dorsal root ganglia, and prefrontal cortex in NP rats compared to sham rats. *C1qb* knockdown alleviated the rat NP and inhibited the NF‐κB signaling pathway.

**Conclusion:**

Four hub genes (*Ptprc*, *C1qb*, *Fcgr2b*, and *Ccl2*) may be potential biomarkers in NP pathogenesis, offering insights into its molecular mechanisms and suggesting therapeutic targets. *C1qb* knockdown is demonstrated to alleviate the NP progression through the NF‐κB signaling pathway.

## Graphical abstract

The differentially expressed genes (DEGs) in neuropathic pain (NP) and their potential regulation by miRNAs were investigated by differential expressionanalysis and protein‐protein interaction network. Fianlly, *Aif1, Ptprc, C1qb, Fcgr2b*, and *Ccl2* emerged as potential biomarkers for NP; miR‐124‐3p, miR‐128‐3p, and miR‐369‐3p may target *Ccl2* and Ptprc to regulate NP.

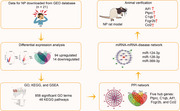

## 1 Introduction

Neuropathic pain (NP) is characterized as persistent pain arising from damage or disorders affecting the somatosensory nervous system (Moisset 2024). Epidemiological statistics indicate that NP affects approximately 7%–10% of people worldwide, accounting for 20%–25% of individuals with chronic pain (Bouhassira 2019; Ahmadi et al. 2024). Patients with NP often experience sleep disturbances, depression, and anxiety, significantly impacting their quality of life (Patel and Dickenson 2022). Consequently, NP has emerged as a major global health problem. There are multiple causes of NP, including peripheral nerve damage, neuromuscular diseases, metabolic disorders, chronic infections, and cancers (Ben Aziz and Cascella 2023). Currently, the treatment methods for NP primarily consist of analgesics and conventional nerve blocks (Gadepalli et al. 2024; Zhang et al. 2024), but their therapeutic effects are limited and accompanied by adverse reactions (Sayo et al. 2019). Hence, it is still crucial to explore the pathogenesis of NP and discover effective treatment strategies.

MicroRNAs (miRNAs) have recently garnered attention as key modulators of gene expression, exerting influential roles in various physiological and pathological processes, including pain (Morchio et al. 2023). Bai et al. (2007) provided the inaugural evidence of the association between pain and miRNAs by identifying seven dysregulated miRNAs in the masseter muscle of rats. Subsequent studies have identified dysregulated miRNA expression patterns in NP and demonstrated the involvement of miRNA in pain‐related signaling pathways (Qi et al. 2022; Yang et al. 2023). For example, Qi et al. (2022) demonstrated that the downregulation of miR‐32‐5p in trigeminal ganglion neurons, mediated by histone methylation, regulates trigeminal NP by targeting Cav3.2 channels. MiRNAs exert their regulatory influences on gene expression posttranscriptionally by targeting messenger RNAs (mRNAs) for degradation or translational repression (Yao et al. 2024). Wang et al. (2023) showed that inhibiting miR‐19a‐3p may alleviate NP by targeting *KLF7* in rats with chronic constriction injury (CCI) (Liu et al. 2023). MiR‐506‐3p attenuates microglial activation via the CCL2/CCR2/NF‐ĸB axis, thereby alleviating NP after brachial plexus avulsion (Jin et al. 2023). These findings demonstrate the complex relationship between miRNA–mRNA interactions and NP.

Given the emerging roles of miRNAs in NP and the limited understanding of the intricate relationships among miRNAs, their target genes, and NP, our study aimed to comprehensively investigate differentially expressed genes (DEGs) in NP and their potential regulation by miRNAs. We screened DEGs in NP using the GSE30691 and GSE18803 databases and performed enrichment analysis to establish protein–protein interaction (PPI) networks and identify hub genes in NP. Additionally, we explored the potential miRNA regulators of these hub genes and subsequently constructed miRNA–mRNA‐disease networks. Considering that different neuropathic models (spared nerve injury [SNI], CCI, spinal nerve ligation [Ch], etc.) showed distinct molecular pathways (Yokoyama et al. 2020), we also investigate the expression differences of the hub genes in different models. This research advances our understanding of the molecular mechanisms of NP and identifies potential miRNA‐based therapeutic targets.

## 2 Materials and Methods

### 2.1 Data Retrieval

Gene expression datasets related to NP were retrieved from the Gene Expression Omnibus (GEO) database (https://www.ncbi.nlm.nih.gov/geo/). Two datasets, GSE30691 (consisting of nine normal samples and nine NP samples) and GSE18803 (consisting of 12 normal samples and 12 NP samples), were selected for further analysis. All the samples were from *Rattus norvegicus*. The NP models used in the GSE30691 dataset included three sciatic nerve lesions: SNI, CCI, and Ch. The NP models used in the GSE18803 dataset were SNI.

### 2.2 Differential Gene Expression Identification

The R limma package (Ritchie et al. 2015) was employed to identify DEGs in the GSE30691 and GSE18803 datasets, with *p* < 0.05.

### 2.3 Gene Ontology (GO) and Kyoto Encyclopedia of Genes and Genomes (KEGG) Enrichment Analysis

To investigate the function and pathways of the DEGs, GO and KEGG pathway enrichment analysis was performed utilizing the R clusterProfiler package (Yu et al. 2012). The significance was set as *p* < 0.05.

### 2.4 Gene Set Enrichment Analysis (GSEA)

GSEA, a method designed to assess the enrichment of predefined gene sets, was employed in this study to analyze the KEGG pathway dataset. Genes were ranked on the basis of their differential expression levels (logFC). Subsequently, predefined gene sets were tested for enrichment at the top (activated pathways) or bottom (inhibited pathways) of this ranked list. GSEA on the DEGs was performed using the R clusterProfiler and GseaVis packages in version 4.2 of R. Significant enrichment was determined with a *p* < 0.05.

### 2.5 PPI Network Construction

To predict protein interactions among the DEGs, we utilized the STRING database (https://cn.string‐db.org/) (Szklarczyk et al. 2021) with a score cutoff >0.4 and a limit <10. The resulting PPI network was established using Cytoscape software. The cytoHubba plugin in Cytoscape was employed to identify hub targets by intersecting the EPC, MCC, MNC, and degree algorithms.

### 2.6 miRNA–mRNA‐Disease Network Analysis

The ENCORI database (https://starbase.sysu.edu.cn/index.php) (Li et al. 2014) was used to predict miRNAs that potentially regulate the identified hub genes. The parameters were set as clipNum = 1, deNum = 0, and proNum = 1. For disease enrichment analysis of the predicted miRNAs, we employed the microRNA enrichment analysis and annotation (miEAA) tool (https://ccb‐compute2.cs.uni‐saarland.de/mieaa2/) (Backes et al. 2016). The significance was set as *p* < 0.05.

### 2.7 Animal Experiments

Adult male Sprague‐Dawley rats, aged 8 weeks and weighing between 220 and 280 g, were selected from Beijing Huafu Kang Biological Technology Co. Ltd (Beijing, China). Rats were accommodated in a specific pathogen‐free facility with controlled environmental conditions, including a temperature range of 20–24°C and a relative humidity of 40%–70%. A 12‐h light–dark cycle was upheld, and the experimental rats had unrestricted access to standard laboratory rodent chow and water. The experiments began after 7‐day adoptive cultivation.

In the first phase of the animal experiments, 27 rats were randomly allocated into three groups (*n* = 9 each group): control, sham, and NP groups. The NP model was established by CCI method following a previous publication (Chang et al. 2022). In the NP group, rats were injected with 50 mg/kg pentobarbital (2%), followed by ligation of the right sciatic nerve at four locations approximately 1 mm apart. The rats in sham group underwent skin and muscle incision and suturing without nerve ligation. The rats in control group received no operation. On the third, seventh, and 14th days after modeling, paw mechanical withdrawal threshold (PMWT) and paw thermal withdrawal latency (PTWL) values (Chang et al. 2022) were assessed to verify the model success. Subsequently, three of the rats at each time point were euthanized by CO_2_ euthanasia followed by cervical dislocation. The blood sample, L3–L5 spinal cord, the dorsal root ganglion (DRG) of the fourth and fifth lumbar vertebrae, and the prefrontal cortex (PFC) tissues were collected on the 0th, third, and 14th days after modeling for subsequent experiments.

In the second phase of the animal experiments, sh‐NC and sh‐C1qb lentivirus were constructed in Designer of Small Interfering RNA website. The sequence of sh‐C1qb was SS sequence: CCCTGTAGATGTTACAGAA and AS sequence: TTCTGTAACATCTACAGGG. A total of 15 rats were randomly allocated into five groups (*n* = 3 in each group): control, sham, NP, NP + sh‐NC, and NP + sh‐C1qb groups. In the NP + sh‐NC and NP + sh‐*C1qb* groups, sh‐NC and NP + sh‐*C1qb* lentivirus (2 × 108 TU/mL) were intrathecally injected into rats in the 3 days before the operation. On the 14th after modeling, the DRG tissue was collected for the subsequent analysis.

### 2.8 Assessment of Rat Behavior

PMWT and PTWL were used for the assessment of rat behavior at nine a.m. on the zero, 3, 7, and 14 days following the principle of blinding. For detection of PMWT, rats were separately placed in the acrylic box (30 × 30 × 30 cm^3^). After a 30‐min adaptation, the rapid foot‐lifting response of rats during the stimulation time or when recorded was regarded as a positive response. Each rat's right hind paw was stimulated three times, with each stimulation given at least 15 s apart, and the response caused by the stimulation (such as licking the foot and swinging the leg) completely disappeared. The average value of the pressure values corresponding to Von‐Frey is taken as PMWT.

For detection of PTWL, rats were separately placed in the acrylic box (7 × 9 × 11 cm^3^). After a 30‐min adaptation, the PTWL was detected. The BME‐410C fully automatic heat pain stimulator was used to irradiate the right hind paw of rats, and the time for the occurrence of leg lifting avoidance was PTWL. Each rat was stimulated three times. An automatic cutoff time of 20 s was set to avoid tissue damage, and the interval between each heat treatment is 5 min.

### 2.9 Determination of Relative Gene Expression of Hub Genes by Quantitative Real‐Time PCR (qRT‐PCR)

RNA extraction was carried out from blood and tissue samples using the RNAeasy Blood RNA Extraction Kit (Beyotime). Subsequently, cDNA synthesis was carried out using the FastKing One‐Step RT‐qPCR Kit (TianGen, Beijing, China). The qRT‐PCR reaction was conducted using SYBR Green PCR Master Mix (Lifeint, Xiamen, China) on the CFX Connect Real‐Time PCR Detection System (Bio‐Rad, CA, USA). The cycling conditions comprised an initial denaturation step at 95°C for t min and 40 cycles of 95°C for 12 s and 62°C for 40 s. The relative expression levels of the target genes were determined using the 2^−ΔΔCt^ method, with GAPDH serving as the internal reference gene. The primer sequences used are listed in Table 1.

**TABLE 1:** Primer sequences for quantitative real‐time PCR (qRT‐PCR) analysis.

Gene	Primer sequences (5′–3′)	Length (bp)
*Aif1* (rat)	Forward: CTCAAATCGTGGCAAGGCTG	95
	Reverse: GGAGAGCAGGACAAAGGACC	
*C1qb* (rat)	Forward: TTCACCTACCACGCCAGTTC	145
	Reverse: GCTTCAAGACTACCCCACCC	
*Ccl2* (rat)	Forward: TAGCATCCACGTGCTGTCTC	94
	Reverse: CAGCCGACTCATTGGGATCA	
*Fcgr2b* (rat)	Forward: TGTGCTAAATCTTGTTGCTGAG	790
	Reverse: TGTTGGGTCCAGTCCAGATG	
*Ptprc* (rat)	Forward: CGACGATGGACTGGACACAA	226
	Reverse: GCTGCTGAGTGTCTGAGTGT	
*GAPDH* (rat)	Forward: GCTGAGAATGGGAAGCTGGT	231
	Reverse: CTCGTGGTTCACACCCATCA	

### 2.10 Determination of Protein Expression of Hub Genes by Western Blotting

The protein levels of C1qb, NF‐ĸB p65, and NF‐ĸB p‐p65 in DRG tissue were detected using western blotting. Samples were treated with radioimmuno‐precipitation assay (RIPA) lysis buffer (P0013B, Beyotime), and then the protein concentration was quantified using a bicinchoninic acid (BCA) kit (P0010S, Beyotime). GAPDH served as the internal control. Primary antibodies used in the study were as follows: C1qb rabbit polyclonal antibody (1:1000, AF7021, Affinity), p65 rabbit polyclonal antibody (1:1000, AF5006, Affinity), p‐p65 rabbit polyclonal antibody (1:1000, AF2006, Affinity), and GAPDH rabbit polyclonal antibody (1:1000, AF7021, Affinity).

### 2.11 Data Analysis

All data are presented as mean ± standard deviation. Statistical analysis was conducted using GraphPad 7.0 software. Normal distribution analysis was conducted using D'Agostino–Pearson, and homogeneity of variance was tested using Bartlett's test. Data that conformed to the normal distribution and homogeneity of variance were compared using one‐ or two‐way analysis of variance for group comparisons. Data that conformed to significance for intergroup comparisons were established at a threshold of *p* < 0.05.

## 3 Results

### 3.1 Identification of DEGs

**FIGURE 1:**
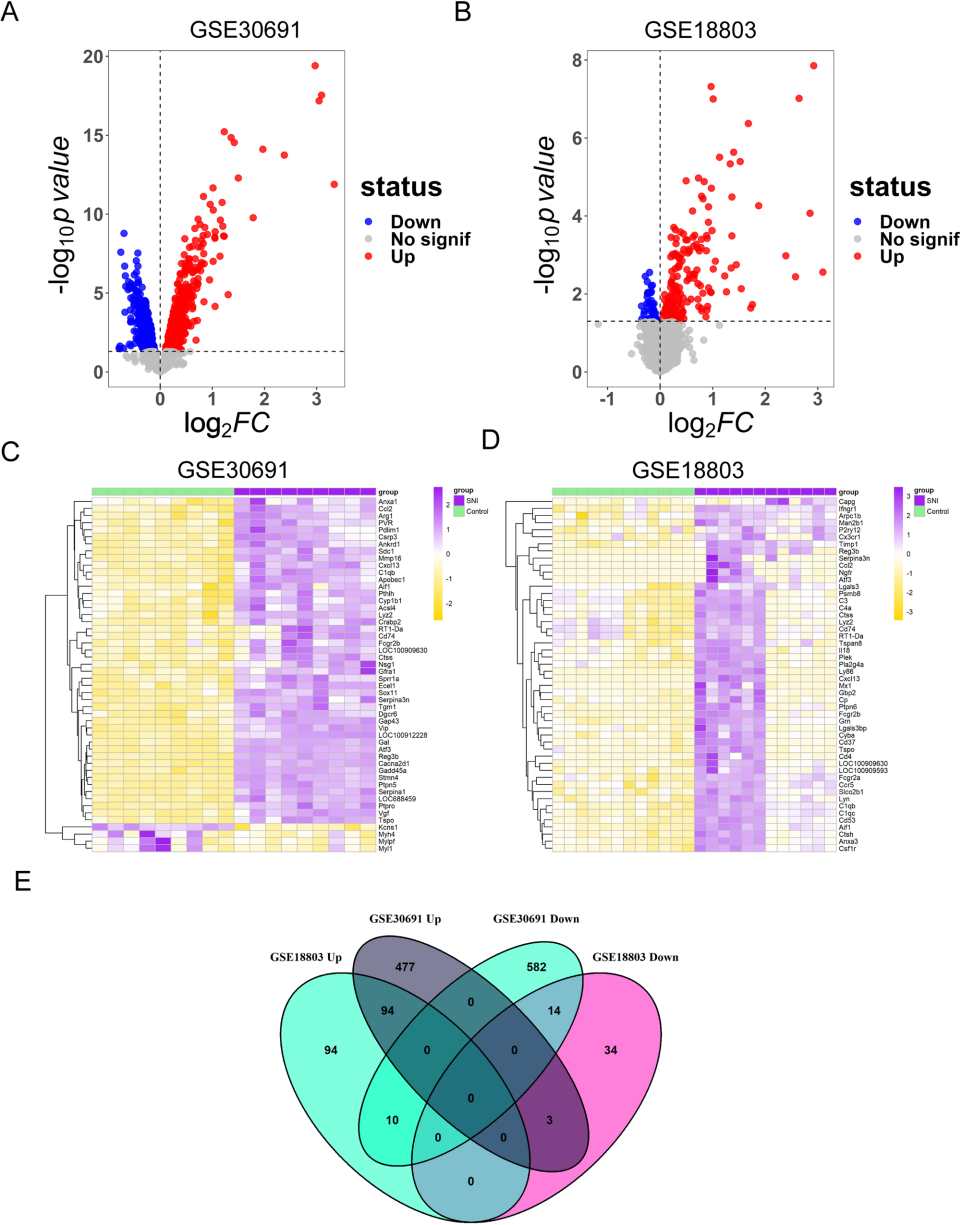
Identification of differentially expressed genes (DEGs). (A and B) Volcano plots depicting the DEGs in the GSE30691 and GSE18803 datasets. (C and D) Heatmaps visualizing the DEGs in the GSE30691 and GSE18803 datasets. (E) Venn diagram showing the intersection of DEGs in the GSE30691 and GSE18803 datasets. SNI, spared nerve injury.

Using the limma R package, a total of 1180 DEGs from the GSE30691 dataset and 249 DEGs from GSE18803 dataset were identified (Figure 1A,B). The heatmap visualizations demonstrated the distinct clustering of DEGs in both datasets (Figure 1C,D). Furthermore, Venn analysis revealed 108 common DEGs (94 upregulated and 14 downregulated) co‐shared in both the two datasets (Figure 1E).

### 3.2 GO and KEGG Enrichment Analysis of DEGs

**FIGURE 2:**
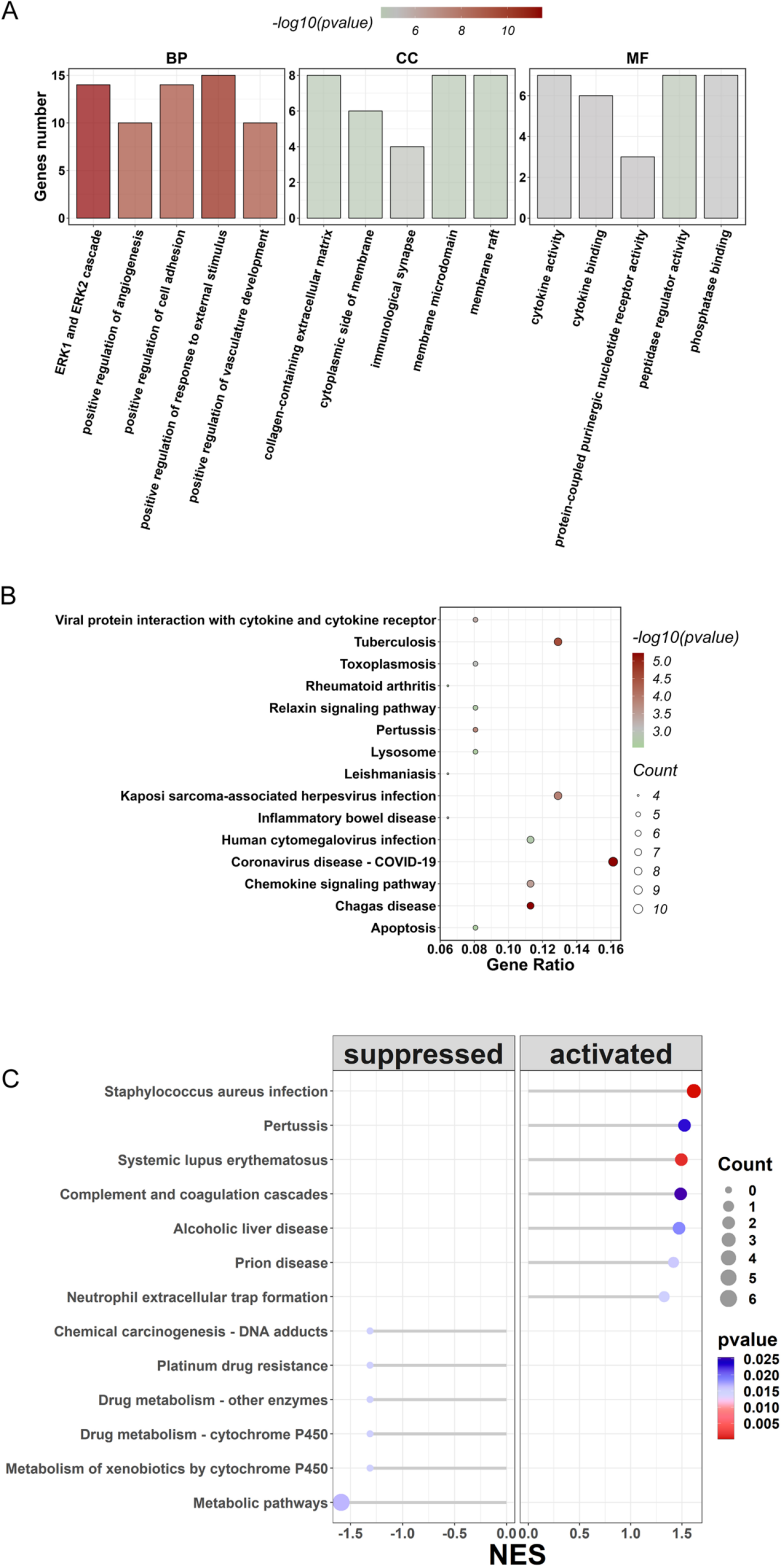
Gene ontology (GO) and Kyoto encyclopedia of genes and genomes (KEGG) enrichment analysis of DEGs. (A) Top 15 significant GO terms (biological process [BP], cellular component [CC], and molecular function [MF]) among the 108 common DEGs. (B) Top 15 enriched KEGG pathways among the 108 common DEGs. (C) Gene set enrichment analysis (GSEA) of 108 common DEGs.

To uncover the functional roles of the 108 common DEGs in NP, we conducted GO and KEGG pathway enrichment analysis. The analysis resulted in a total of 858 significant GO terms and 46 KEGG pathways (*p* < 0.05). The top 15 significant GO terms (Figure 2A) encompassed various biological processes (ERK1 and ERK2 cascade, cell adhesion, positive regulation of angiogenesis, response to external stimulus, and vasculature development), cellular components (collagen‐containing extracellular matrix, cytoplasmic side of the membrane, immunological synapse, membrane microdomain, and membrane raft), and molecular functions (cytokine activity, peptidase regulator activity, and phosphatase binding). Additionally, the KEGG results showed that the 108 common DEGs in NP were related to inflammatory‐related pathways like viral protein interaction with cytokine and cytokine receptor and inflammatory bowel disease (Figure 2B).

### 3.3 GSEA Results

Subsequently, GSEA analysis was conducted on the 108 common DEGs using the clusterProfiler and GseaVis packages. The 108 DEGs were significantly enriched into 13 signaling pathways (*p* < 0.05). Notably, *Staphylococcus aureus* infection, complement and coagulation cascades, and neutrophil extracellular trap formation displayed activation patterns, whereas metabolic pathways, metabolism of xenobiotics, and drug metabolism‐cytochrome P450 by cytochrome P450 exhibited inhibition regulation (Figure 2C).

### 3.4 PPI Network Construction and Identification of Hub Genes

**FIGURE 3:**
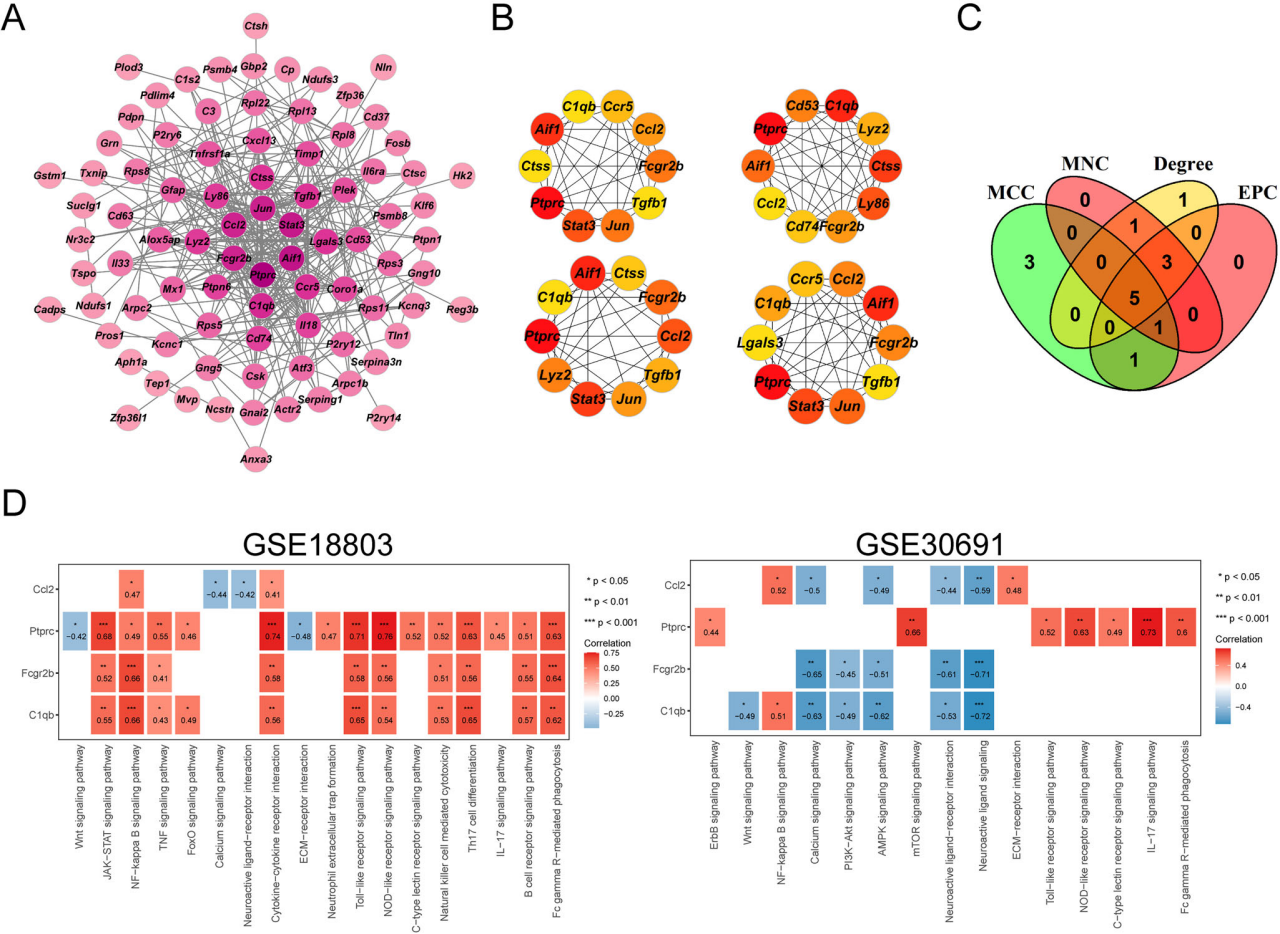
Protein–protein interaction (PPI) network construction and hub gene screening. (A) PPI network of the 108 common DEGs. (B) Top 10 hub genes selected from the PPI network using degree, EPC, MCC, and MNC algorithms (from left to right). (C) Intersection of degree, EPC, MCC, and MNC algorithms to identify hub genes. (D) The correlations of the five hub genes with the inflammatory pathways.

Using the STRING database, we predicted protein interactions among the 108 common DEGs, resulting in a PPI network comprising 86 nodes and 321 edges (Figure 3A). Subsequently, the top 10 hub genes were identified using the cytoHubba plugin based on degree, EPC, MCC, and MNC algorithms (Figure 3B). The intersection of these algorithms led to the identification of five hub genes (*Ptprc*, *C1qb*, *Aif1*, *Fcgr2b*, and *Ccl2*) (Figure 3C). The five hub genes were demonstrated to significantly correlate with the immune and inflammatory response‐related pathways, especially the NF‐κB signaling pathway (Figure 3D).

### 3.5 miRNA–mRNA‐Disease Network Analysis

**FIGURE 4:**
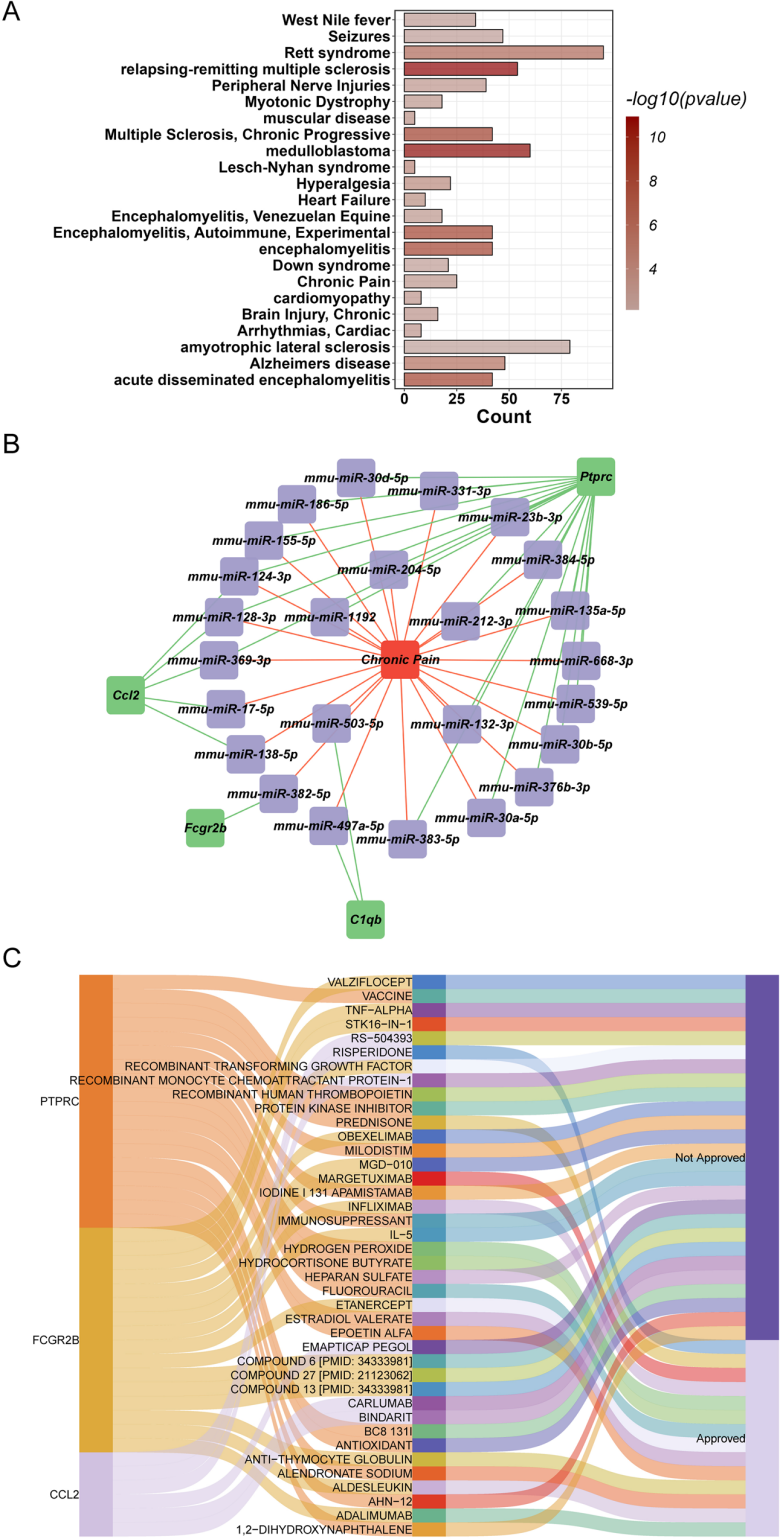
miRNA–mRNA‐disease network analysis. (A) Enrichment analysis of miRNA‐associated diseases. (B) miRNA–mRNA‐chronic pain network visualization. (C) Drug prediction for the hub genes.

Further analysis was conducted in ENCORI database, and potential miRNAs regulating the five hub genes (*Ptprc*, *C1qb*, *Aif1*, *Fcgr2b*, and *Ccl2*) were explored, and a total of 160 associated miRNAs were confirmed to regulate the five hub genes. To assess the disease relevance of these miRNAs, we performed a disease enrichment analysis using the miEAA tool. The results indicated that these miRNAs were involved in 23 diseases, including chronic pain (Figure 4A). Subsequently, we constructed a miRNA–mRNA‐chronic pain network using Cytoscape software, which consisted of 30 nodes and 53 edges (Figure 4B). Among the identified miRNAs, 25 of which were found to regulate four hub genes (*Ptprc*, *C1qb*, *Fcgr2b*, and *Ccl2*) in chronic pain. Notable miRNAs involved in this regulation include miR‐124‐3p, miR‐128‐3p, and miR‐369‐3p, which were related to at least two genes (Table 2). These evidences indicated that these hub genes might be the important targets in NP. The drug prediction indicated that these hub genes were significantly correlated with 40 small molecule drugs, like hydrogen peroxide, alendronate sodium, and risperidone (Figure 4C).

**TABLE 2:** The list of miRNA–mRNA‐chronic pain network.

miRNA name	mRNA	Disease	miRNA name	mRNA	Disease
mmu‐miR‐503‐5p	*C1qb*	Chronic pain	mmu‐miR‐135a‐5p	*Ptprc*	Chronic pain
mmu‐miR‐497a‐5p	*C1qb*	Chronic pain	mmu‐miR‐155‐5p	*Ptprc*	Chronic pain
mmu‐miR‐124‐3p	*Ccl2*	Chronic pain	mmu‐miR‐186‐5p	*Ptprc*	Chronic pain
mmu‐miR‐128‐3p	*Ccl2*	Chronic pain	mmu‐miR‐204‐5p	*Ptprc*	Chronic pain
mmu‐miR‐138‐5p	*Ccl2*	Chronic pain	mmu‐miR‐30d‐5p	*Ptprc*	Chronic pain
mmu‐miR‐17‐5p	*Ccl2*	Chronic pain	mmu‐miR‐331‐3p	*Ptprc*	Chronic pain
mmu‐miR‐369‐3p	*Ccl2*	Chronic pain	mmu‐miR‐212‐3p	*Ptprc*	Chronic pain
mmu‐miR‐382‐5p	*Fcgr2b*	Chronic pain	mmu‐miR‐383‐5p	*Ptprc*	Chronic pain
mmu‐miR‐23b‐3p	*Ptprc*	Chronic pain	mmu‐miR‐376b‐3p	*Ptprc*	Chronic pain
mmu‐miR‐30a‐5p	*Ptprc*	Chronic pain	mmu‐miR‐539‐5p	*Ptprc*	Chronic pain
mmu‐miR‐30b‐5p	*Ptprc*	Chronic pain	mmu‐miR‐369‐3p	*Ptprc*	Chronic pain
mmu‐miR‐124‐3p	*Ptprc*	Chronic pain	mmu‐miR‐668‐3p	*Ptprc*	Chronic pain
mmu‐miR‐128‐3p	*Ptprc*	Chronic pain	mmu‐miR‐384‐5p	*Ptprc*	Chronic pain
mmu‐miR‐132‐3p	*Ptprc*	Chronic pain	mmu‐miR‐1192	*Ptprc*	Chronic pain

### 3.6 Hub Genes Expression

**FIGURE 5:**
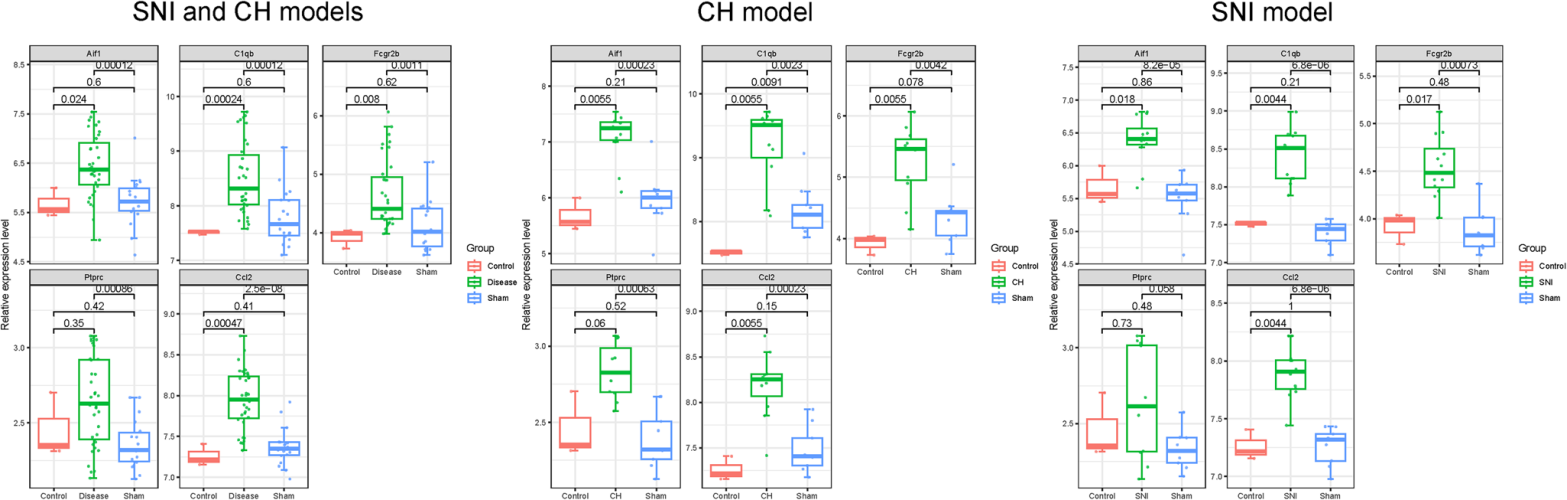
Expression analysis of Aif1, Ptprc, C1qb, Fcgr2b, and Ccl2 in different NP models in datasets. SNI, spared nerve injury.

Expression analysis of five hub genes, *Aif1*, *Ptprc*, *C1qb*, *Fcgr2b*, and *Ccl2*, was conducted in GSE30691 and GSE18803 datasets. The results revealed upregulated expression levels of these genes in NP samples (SNI and CH models) compared to control samples (Figure 5).

**FIGURE 6:**
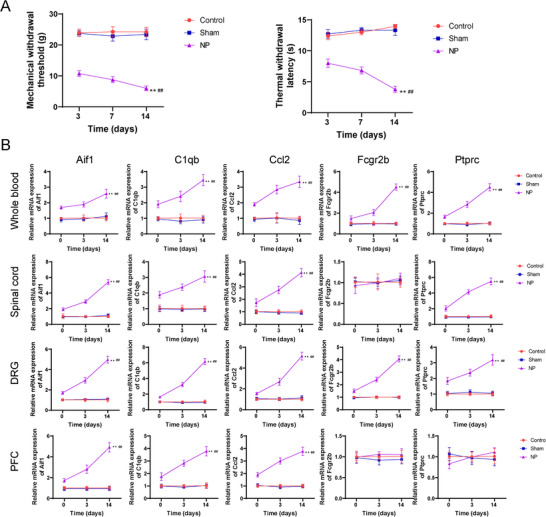
The expression of *Aif1*, *Ptprc*, *C1qb*, *Fcgr2b*, and *Ccl2* in NP rat model. (A) Validation of the NP rat model through assessment of PMWT (paw mechanical withdrawal threshold) and PTWL (paw thermal withdrawal latency) values. (B) Relative mRNA expression levels of *Aif1*, *Ptprc*, *C1qb*, *Fcgr2b*, and *Ccl2* in NP rats compared to the control group. *p < 0.05, **p < 0.01 versus control group; #*p* < 0.05, ##*p* < 0.01 versus sham group. DRG, dorsal root ganglion; mRNA, messenger RNA; NP, neuropathic pain; PFC, prefrontal cortex.

Furthermore, we established an NP rat model by CCI method to validate the findings from the bioinformatics analysis. PMWT and PTWL values were markedly reduced in the NP group compared to the sham group on the third, fifth, and seventh days after modeling (*p* < 0.01; Figure 6A), indicating the successful establishment of the NP rat model. Additionally, the NP group exhibited a significant increase in the relative mRNA expression levels of *Aif1*, *Ptprc*, *C1qb*, *Fcgr2b*, and *Ccl2* compared to the control group in the blood, spinal cord, DRG, and PFC tissues (*p* < 0.05; Figure 6B), which were coincident with the results from GEO datasets. Moreover, the expression levels of *Aif1*, *Ptprc*, *C1qb*, *Fcgr2b*, and *Ccl2* were all significantly negatively correlated with the PMWT and PTWL values (Table 3).

**TABLE 3:** Correlations between each key gene with rat behavior.

Sample type	Rat behavior	Key genes	Correlation index	*R* ^2^	*p* value
Whole blood	PMWT	*Aif1*	−11.890	0.832	<0.001
		*C1qb*	−7.654	0.842	<0.001
		*Ccl2*	−7.478	0.850	<0.001
		*Fcgr2b*	−13.520	0.782	<0.001
		*Ptprc*	−5.478	0.759	<0.001
	PTWL	*Aif1*	−5.774	0.878	<0.001
		*C1qb*	−3.707	0.882	<0.001
		*Ccl2*	−3.574	0.868	<0.001
		*Fcgr2b*	−6.315	0.762	<0.001
		*Ptprc*	−2.723	0.838	<0.001
Spinal cord	PMWT	*Aif1*	−4.448	0.737	<0.001
		*C1qb*	−8.865	0.842	<0.001
		*Ccl2*	−6.009	0.773	<0.001
		*Fcgr2b*	0.028	2.430^−7^	>0.05
		*Ptprc*	−4.120	0.820	<0.001
	PTWL	*Aif1*	−2.264	0.853	<0.001
		*C1qb*	−4.303	0.887	<0.001
		*Ccl2*	−3.005	0.864	<0.001
		*Fcgr2b*	−1.269	0.002	>0.05
		*Ptprc*	−2.006	0.869	<0.001
DRG	PMWT	*Aif1*	−4.899	0.746	<0.001
		*C1qb*	−3.660	0.670	<0.001
		*Ccl2*	−6.287	0.736	<0.001
		*Fcgr2b*	−4.575	0.680	<0.001
		*Ptprc*	−8.859	0.848	<0.001
	PTWL	*Aif1*	−2.447	0.832	<0.001
		*C1qb*	−1.850	0.799	<0.001
		*Ccl2*	−2.313	0.777	<0.001
		*Fcgr2b*	−3.153	0.827	<0.001
		*Ptprc*	−4.303	0.894	<0.001
PFC	PMWT	*Aif1*	−4.827	0.727	<0.001
		*C1qb*	−6.685	0.812	<0.001
		*Ccl2*	−6.701	0.851	<0.001
		*Fcgr2b*	−16.29	0.063	>0.05
		*Ptprc*	0.208	1.718^−5^	>0.05
	PTWL	*Aif1*	−2.428	0.822	<0.001
		*C1qb*	−3.249	0.858	<0.001
		*Ccl2*	−3.267	0.905	<0.001
		*Fcgr2b*	−8.344	0.073	>0.05
		*Ptprc*	−1.552	0.004	>0.05

Abbreviations: DRG, dorsal root ganglion; PFC, prefrontal cortex; PMWT, paw mechanical withdrawal threshold; PTWL, paw thermal withdrawal latency.

### 3.7 C1qb Knockdown Alleviated the NP

**FIGURE 7:**
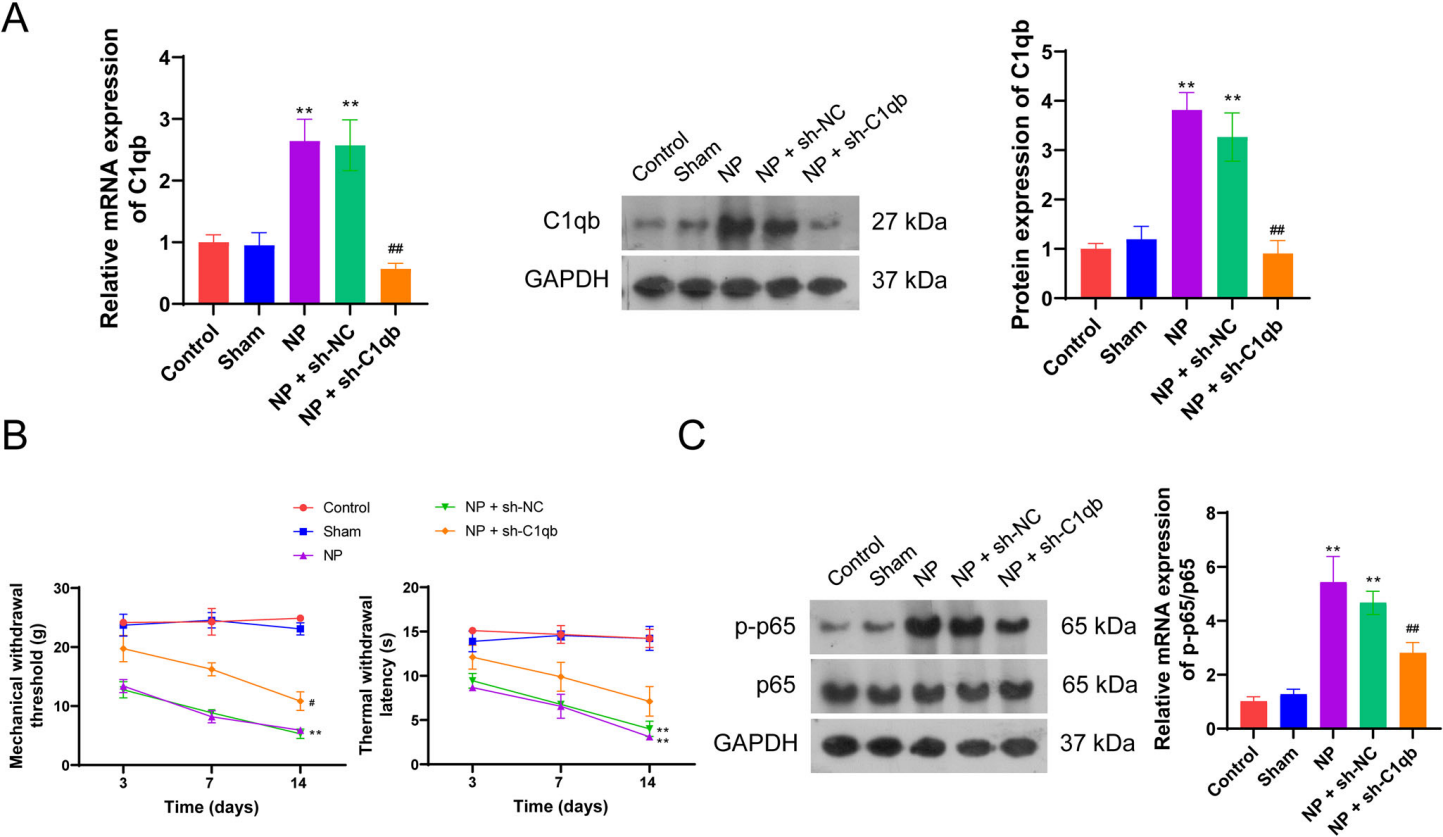
*C1qb* knockdown alleviated the NP performance. (A) Expression analysis of *C1qb* detected by qRT‐PCR and western blotting. (B) Assessment of PMWT (paw mechanical withdrawal threshold) and PTWL (paw thermal withdrawal latency) values. (C) The expression levels of NF‐κB signaling pathway proteins p‐p65/p65 detected by western blotting. *p < 0.05, **p < 0.01 versus sham group; ^#^ *p* < 0.05, ^##^ *p* < 0.01 versus NP + sh‐NC group. mRNA, messenger RNA; NP, neuropathic pain.

To further investigate the crucial role of hub genes in NP, *C1qb* knockdown treatment was conducted (Figure 7A). With the *C1qb* knockdown, the rat pain behaviors exhibited some alleviation (Figure 7B). Moreover, inflammatory‐related NF‐κB signaling pathway proteins p‐p65/p65 were also decreased by *C1qb* knockdown (Figure 7C).

## 4 Discussion

Chronic pain is a complex condition characterized by persistent pain signals in the absence of ongoing tissue damage (Hylands‐White et al. 2017). NP is a subtype of chronic pain that severely affects people's quality of life and work. Currently, clear biomarkers for the NP treatment are absent, and the management for NP remains inadequate (Uniyal et al. 2023; Sharma et al. 2018); therefore, understanding the molecular basis of NP is imperative for the formulation of effective therapeutic strategies. This study employed biochemical techniques to investigate the molecular mechanisms of NP. We identified 108 common DEGs between NP and normal tissues from the GSE30691 and GSE10883 datasets, comprising 94 upregulated and 14 downregulated genes, suggesting their potential roles as regulators in NP.

To understand the functional implications of the 108 common DEGs, we conducted GO and KEGG pathway enrichment analyses. Our findings revealed significant GO terms linked to NP, including the ERK1 and ERK2 cascade, positive regulation of angiogenesis, and cell adhesion, affirming previous studies that associated these biological processes with pain pathophysiology (Li et al. 2015; Sharma et al. 2023; Sakai et al. 2008). Moreover, the implicated roles of cytokine activity and cytokine binding underscore the contribution of immune‐inflammatory components to NP (Scarneo et al. 2023; Mollazadeh et al. 2019). Furthermore, the recognition of the collagen‐containing extracellular matrix and membrane microdomain as key cellular components corresponds with the acknowledged roles of extracellular matrix remodeling and lipid rafts in neuronal plasticity and pain sensitization (Miller et al. 2020). The enriched KEGG pathways in our study provide insights into the molecular pathways associated with NP. Growing evidence suggests that targeting chemokine signaling pathways may be critical for regulating NP (Brandolini et al. 2019; Guo et al. 2020; Chang and Zhao 2022). For example, protocatechuic acid alleviates NP in CCI rats by inhibiting JNK/CXCL1/CXCR2 signaling pathway (Chang and Zhao 2022). GO and KEGG enrichment analyses propose a relationship between NP and immune and inflammatory responses.

The GSEA results showcased activation of pathways tied to *S. aureus* infection, complement and coagulation cascades, and neutrophil extracellular trap formation. These findings echo the previous study underscoring the role of innate immune responses and neuroinflammation in NP (Ye et al. 2022). The inhibition of metabolic pathways, particularly drug and xenobiotics metabolism via cytochrome P450, suggests potential disturbances in drug metabolism and detoxification processes in NP (Coluzzi et al. 2022).

In our study, *Ptprc*, *C1qb*, *Aif1*, *Fcgr2b*, and *Ccl2* emerged as hub genes linked with NP. To investigate the expression levels of them in the NP progression, we established a CCI rat model by injection of pentobarbital (2%, 50 mg/kg) followed by ligation of the right sciatic nerve at four locations, which is the same as the Chang et al. (2022) modeling method. Following, the expression levels of *Aif1*, *Ptprc*, *C1qb*, *Fcgr2b*, and *Ccl2* were investigated in the blood, the spinal cord, DRG, and PFC tissues, showing an increase in NP rats compared to sham rats in these different samples. *Ptprc*, Protein Tyrosine Phosphatase Receptor Type C, is also called *CD45*. The specific roles of *Ptprc* have not been reported in NP. A previous study only demonstrated that the expression level of *Ptprc* is upregulated in NP animals (Galbavy et al. 2015), which is coincidence with our results both in the dataset predicting and the animal experiment. The current acknowledgements of *Ptprc* in nervous system are mainly based on microglia. A study demonstrated that *Ptprc* serves as a positive regulator of T cell coactivation, showing upregulation in microglia obtained from LPS‐injected mice (Sousa et al. 2018). Such activated microglia are central to the evolution and perpetuation of NP (Yu et al. 2020; Olson 2010). *C1qb*, a component of the complement system, is involved in diverse responses, including leukocyte and lymphocyte‐mediated immunity. Previous studies clarified the elevated expression of *C1qb* in brain development and brain lesioning (Johnson et al. 1994; Pasinetti et al. 1992). Notably, *C1qb* has been suggested as a predictive or prognostic marker for NP (Yang et al. 2018). In the previous bioinformatics analysis, *C1qb* tends to be upregulated in the NP rats (Wang et al. 2020; Yang et al. 2018), which agrees with our results. In the following investigation for *C1qb*, our results further indicated that *C1qb* knockdown significantly alleviated the NP symptom in CCI rat model, along with inhibition on the NF‐κB signaling pathway. These results indicated the potential of *C1qb* as a target in NP. As for *Fcgr2b*, the existing evidences also emphasized that *Fcgr2b* is intricately linked with the anterior cingulate cortex post‐nerve injury (Zhang et al. 2022). A transcriptomic analysis indicated increased expression of *Fcgr2b* after NP model construction (Zhang et al. 2022), which is also insistent with our results. Several studies have indicated upregulation of *Ccl2* expression in damaged DRG cells following nerve injury. This *Ccl2* upregulation can provoke microglial activation, thereby inducing NP (Smith 2010; Thacker et al. 2009; Jin et al. 2023). The inhibition of *Ccl2* has been shown to effectively alleviate BPA‐induced NP (Xian et al. 2020). All the above evidences demonstrate that the five genes selected from our analysis are related to immune cell activation, neuroinflammation regulation, and chemotaxis, which might exhibit an important role in the progression of NP.

miRNAs have been implicated to be key modulators of gene expression, exerting influential roles in various physiological and pathological processes, including pain (Morchio et al. 2023). Hence, in the present study, miRNA–mRNA‐disease regulation network was constructed. Finally, a total of 25 miRNAs might exert regulatory influence on four of the hub genes: *Ptprc*, *C1qb*, *Fcgr2b*, and *Ccl2*. The apparent dysregulation of these miRNAs in chronic pain suggests their potential utility as therapeutic targets for modulating pain pathways. Among these 25 miRNAs, three (miR‐124‐3p, miR‐128‐3p, and miR‐369‐3p) exerted to be related to at least two genes (*Ptprc* and *Ccl2*). Previous research studies have demonstrated that miR‐124‐3p can attenuate the development of NP through targeting various genes like *EZH2* (Zhang et al. 2019), *Egr1* (Jiang et al. 2021), and *JAG1* (Li et al. 2020). Additionally, miR‐128‐3p has been shown to attenuate the progression of NP by regulating *ZEB1* (Zhang et al. 2020). In our predictive analysis, miR‐124‐3p and miR‐128‐3p appeared capable of targeting *Ccl2* and *Ptprc* to regulate NP, but the regulatory relationships are necessary to be validated in future. As for miR‐369‐3p, the regulatory role in NP has not been reported. However, some studies indicated that miR‐369‐3p regulates tumor progression and inflammatory processes through targeting specific genes (Li et al. 2019; Liu et al. 2019; Scalavino et al. 2020, 2023; Xu and Liu 2020; Yi et al. 2022; Zhao et al. 2021). In our prediction, miR‐369‐3p also exhibited targeting roles on *Ccl2* and *Ptprc* in NP. On the basis of this evidence, we speculated that miR‐369‐3p might also exert an important role in NP.

Our study innovatively demonstrates that the potential mechanism of NP may relate to some key genes (*Aif1*, *Ptprc*, *C1qb*, *Fcgr2b*, and *Ccl2*) and miRNAs (miR‐124‐3p, miR‐128‐3p, and miR‐369‐3p). Nevertheless, limitations exist. First, the sample size in our analysis is too small (only 21 samples in two datasets) and heterogenetic (different NP models in two datasets). Therefore, larger sample size and more cohorts are necessary for the validation in the future. Second, in both bioinformatics analysis and experimental validation, our study only focused on animals. The analysis results needed to be validated in various samples, including blood and tissues (neural tissues in pain pathway like injured nerve, DRG, spinal cord, and supraspinal structures) from animal and human. Third, the study is still inadequate in that the NP models employed young adults, whereas the chronic NP is more prevalent in old adults. Fourth, different NP models might exhibit different molecular pathways (Yokoyama et al. 2020), and how these genes participate in different NP models is necessary to conduct in the future. Finally, the regulatory roles of key genes and miRNAs have not been clarified in our results, and they are needed to be validated in more experiments. Although there are many limitations in this study, the analysis provides a direction for future mechanism exploration in NP.

## 5 Conclusion

In conclusion, our analysis revealed 108 DEGs in the NP datasets, among which *Aif1*, *Ptprc*, *C1qb*, *Fcgr2b*, and *Ccl2* emerged as potential biomarkers for NP. miR‐124‐3p, miR‐128‐3p, and miR‐369‐3p may target *Ccl2* and *Ptprc* to regulate NP, presenting new opportunities for subsequent NP treatment strategies. Our findings are based on database analyses, emphasizing the imperative need for rigorous experimental validation. Subsequent in‐depth functional studies are required to elucidate the exact roles of these identified genes and miRNAs in the intricate mechanisms underlying NP.

## Author Contributions

**Lingqi Yao**: conceptualization, data curation, formal analysis, writing – original draft. **Ming Liu**: conceptualization, formal analysis, writing – review and editing.

## Ethics Statement

All animal experimental procedures were sanctioned by the Experimental Animal Ethics Committee of Xiamen University (XMULAC20220034‐24).

## Conflicts of Interest

The authors declare no conflicts of interest.

## Peer Review

The peer review history for this article is available at https://publons.com/publon/10.1002/brb3.70812.

## Data Availability

The datasets used and/or analyzed during the current study are available from the corresponding author on reasonable request.
